# A new case of 17p13.3p13.1 microduplication resulted from unbalanced translocation: clinical and molecular cytogenetic characterization

**DOI:** 10.1186/s13039-021-00562-1

**Published:** 2021-08-31

**Authors:** Zhanna G. Markova, Marina E. Minzhenkova, Lyudmila A. Bessonova, Nadezda V. Shilova

**Affiliations:** Research Centre for Medical Genetics, Moskvorechye St., 1, Moscow, Russia 115522

**Keywords:** 17p13.3p13.1 microduplication, Chromosomal microarray analysis, FISH

## Abstract

Copy number gain 17 p13.3p13.1 was detected by chromosomal microarray (CMA) in a girl with developmental/speech delay and facial dysmorphism. FISH studies made it possible to establish that the identified genomic imbalance is the unbalanced t(9;17) translocation of maternal origin. Clinical features of the patient are also discussed. The advisability of using the combination of CMA and FISH analysis is shown. Copy number gains detected by clinical CMA should be confirmed using FISH analysis in order to determine the physical location of the duplicated segment. Parental follow-up studies is an important step to determine the origin of genomic imbalance. This approach not only allows a most comprehensive characterization of an identified chromosomal/genomic imbalance but also provision of an adequate medical and genetic counseling for a family taking into account a balanced chromosomal rearrangement.

## Background

Introduction of molecular cytogenetic methods into clinical practice, such as CMA, has become a new stage in the in the genetic diagnosis of human chromosomal abnormalities. This technique allows to identify a copy number variations (CNV), establishing their size, boundaries and genes involved. The data obtained have made it possible to describe genomic disturbances leading to specific clinical phenotypes and identify a novel microdeletion and microduplication syndromes.

Human chromosome 17 is a small chromosome rich in genes linked with a number of well-known microdeletion and microduplication syndromes. Over 23% of the chromosome 17 short arm consist of low-copy repeats, which creates the possibility of a non-allelic homologous recombination [1]. The genomic instability of chromosome 17 promotes development of a wide range of clinical manifestations including cerebral morphological brain abnormalities, mental retardation, epilepsy and tumors [2–4].

The first cases of 17p13.3 microduplication were reported in 2009 [3]. Accumulation of similar cases led later to formation of 17p13.3 microduplication syndrome (OMIM #613215). This is a rare syndrome wherein the critical region overlaps the deletion region in Miller-Dieker Lissencephaly syndrome (OMIM #247200) involving *PAFAH1B1* and/or *YWHAE* genes on chromosome 17p13.3. The identified cases of 17p13.3 microduplication with various sizes and gene contents have different mechanisms of occurrence and different clinical phenotypes. The common clinical features associated with the disease are a mild or moderate psychomotor retardation, hypotonia, abnormalities of hands and feet, cranio-facial dysmorphism including a high forehead with frontal bossing, hypertelorism, a small nose and a small mouth [3, 5, 6].

In this study we report the clinical and molecular cytogenetic characterization of a new case with duplication of chromosome 17 region p13.3p13.1.

## Methods

The CytoScan HD array (Affymetrix, USA) was applied to detect the CNV across the entire genome following the manufacturer’s protocols. Microarray-based copy number analysis was performed using the Chromosome Analysis Suite software version 4.0 (Thermo Fisher Scientific Inc.) and the results were presented on the International System for Human Cytogenomic Nomenclature 2016 (ISCN, 2016). Detected CNVs were totally assessed by comparing them with published literature and the public databases: Database of Genomic Variants (DGV) (http://dgv.tcag.ca/dgv/app/home), DECIPHER (http://decipher.sanger.ac.uk/) and OMIM (http://www.ncbi.nlm.nih.gov/omim). Genomic positions refer to the Human Genome February 2009 assembly (GRCh37/hg19).

FISH was carried out using chromosomal preparations from cultured peripheral blood lymphocytes following the manufacturers’ protocols. DNA probes for subtelomeric regions of the short arm of chromosomes 17, the short arm of chromosome 9, centromere region of chromosome 17, pericentromeric heterochromatin of chromosome 9 (Sub-telomere 17pter, Sub-telomere 9pter, SE 17 (D17Z1), SE 9 (classical); KREATECH), whole chromosome probes for the short and long arms of chromosome 17 (XCAP 17 short, XCAP17 long, KREATECH) were applied.

The analysis was carried out using an AxioImager M.1 epifluorescence microscope (Carl Zeiss) and an Isis digital image processing computer program (MetaSystems).

## Case presentation

The patient, a girl 2 years 3 months old, was referred for CGH due to delayed psychomotor and speech development. Pedigree burdened, mother's brother has mental retardation.

The girl is the first child of healthy non-consanguineous parents. Pregnancy proceeded with the threat of termination, premature aging of the placenta and oligohydramnios from 25 weeks, isthmic-cervical insufficiency (cervical pessary from 30 weeks). Childbirth at 38–39 weeks, weight 3350 g (+ 0.14 SD), height 52 cm (+ 1.45 SD), head circumference 35 cm, chest circumference 34 cm, Apgar score was 7/8, multiple stigmas of dysembryogenesis at birth—broad nasal bridge, telangiectasia of the frontal region, widely spaced nipples, a short lingual frenum.

The child had in the neonatal period a syndrome of central nervous system depression, feeding difficulties, cardiopathy (clinically unimportant patent foramen ovale 3 mm), cephalohematomas of the parietal bones, intrauterine infection (conjunctivitis). She had no clinical or electrographic seizures. Magnetic resonance imaging (MRI) of the brain was not performed.

Her psychomotor development was retarded: holds his head from 2 months, turns over from 10 months, sat independently at 10 months of age, walked without support at 19 months, and articulates about 10 words at the time of examination.

**Fig. 1:**
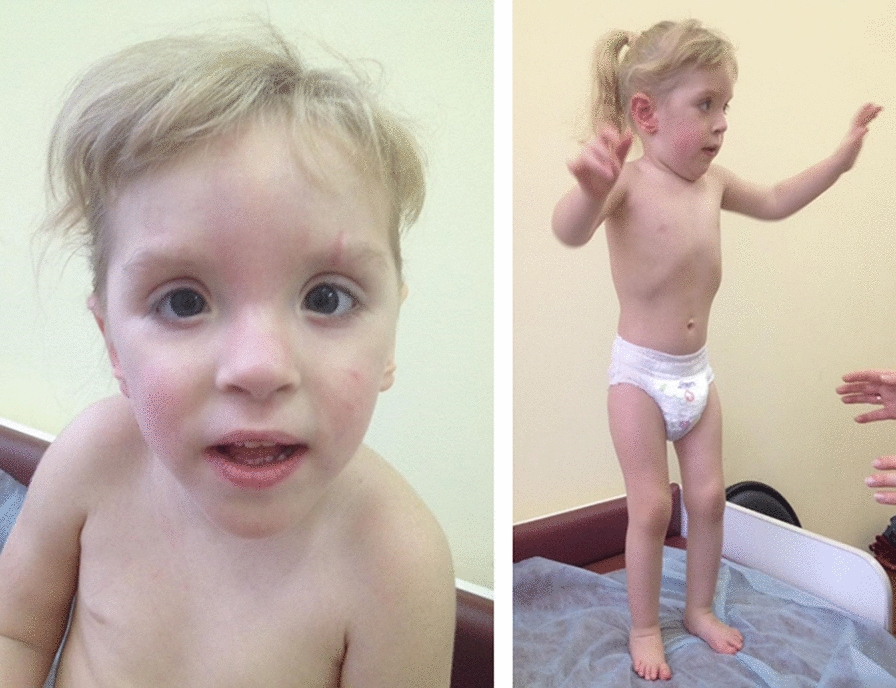
Clinical presentation of the patient

Physical examination: height 88 cm (+ 0.73 SD), weight 12 kg (− 0.70 SD), head circumference 47 cm (− 1.12 SD), decrease in the increase in head circumference to height, frontal bossing, hypertelorism, low-set fissures, small mouth, triangular chin, flexion contractures of the elbow and knee joints, moderate contractures of the fingers, planovalgus feet (Fig. 1).

## Results

**Fig. 2:**
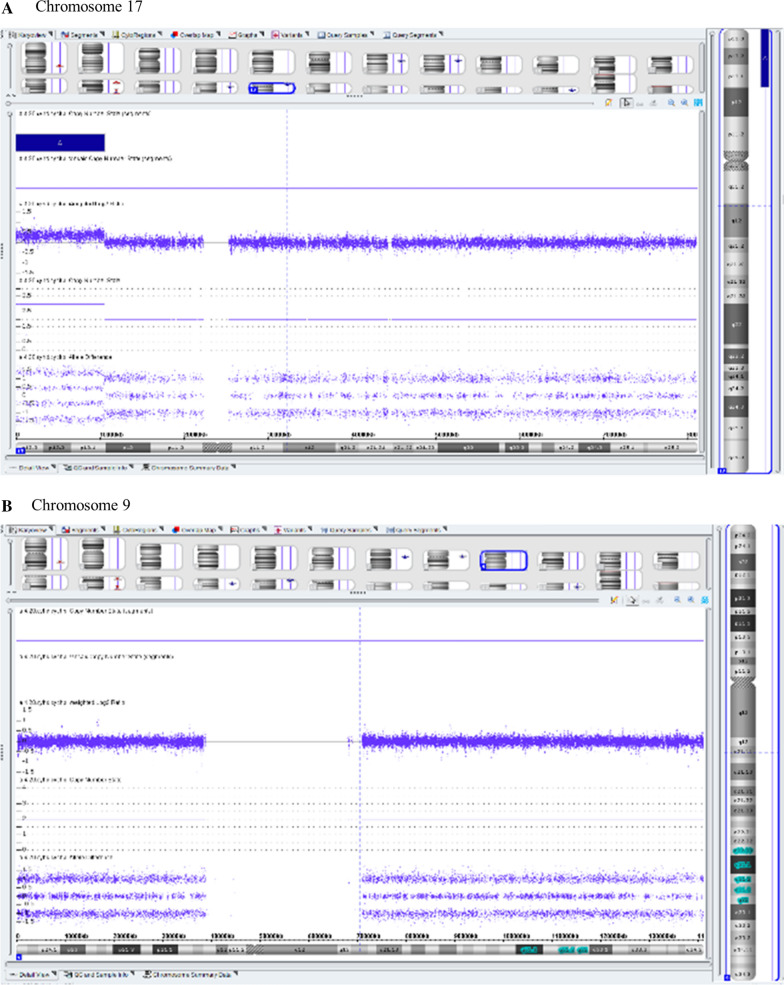
CMA results showing about 10.5 Mb duplication of the 17p subterminal region (**a**) and a normal result of the chromosome 9 (**b**)

CMA revealed a 10.5 Mb pathogenic duplication in terminal region of chromosome 17 affected 285 genes, 182 of which are known OMIM-morbid genes (Fig. 2a). The molecular karyotype of the proband (according to ISCN 2016) was thus: arr[hg19] 17p13.3p13.1(525_10512077)×3.

**Fig. 3:**
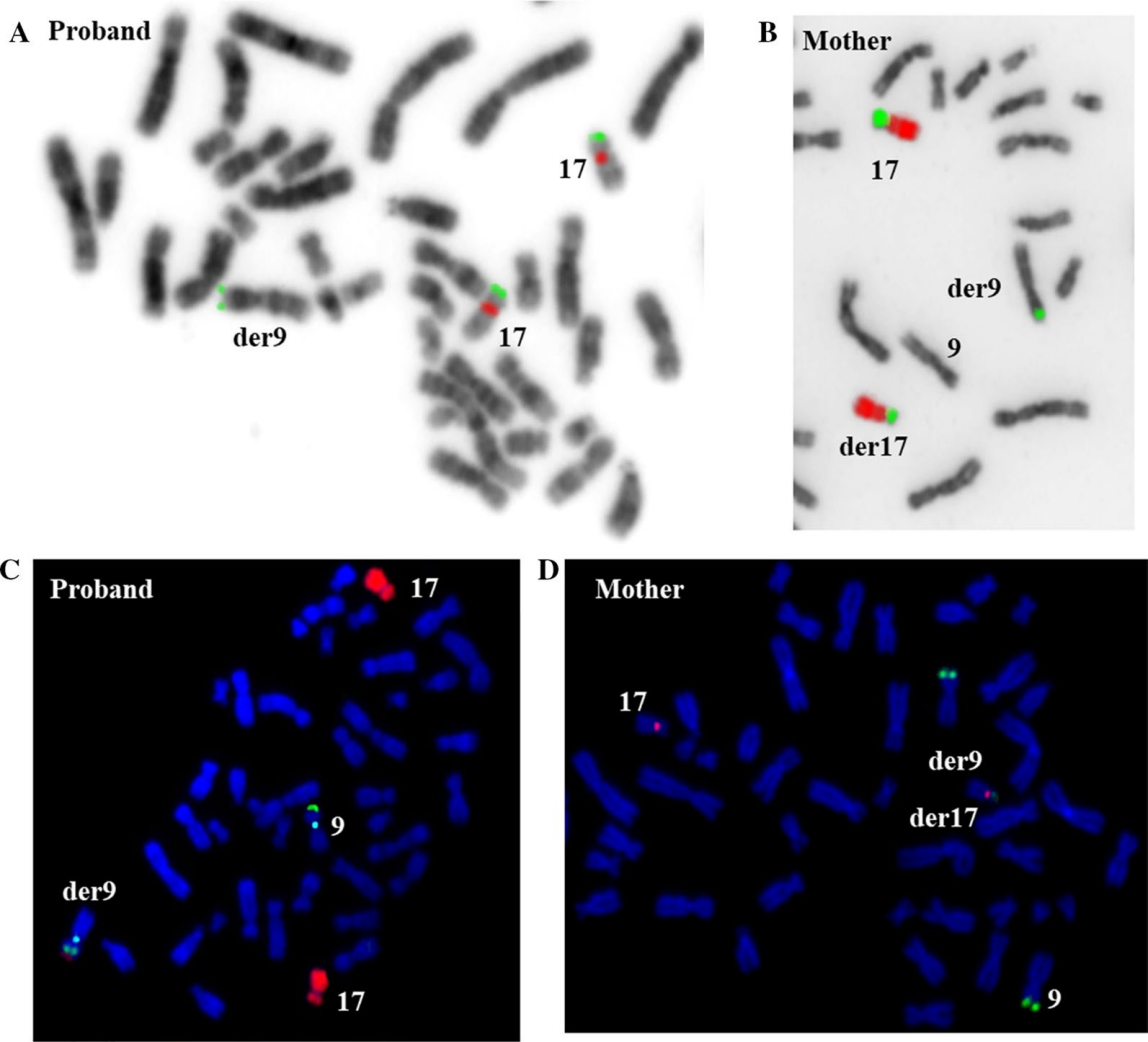
FISH results. **a** FISH analysis with the chromosome 17 subtelomeric (17pter, green) and chromosome 17 centromeric (red) probes. **b** FISH analysis with a partial chromosome painting for short (green) and long (red) arms of chromosome 17. **c** FISH analysis with WCP of chromosome 17, chromosome 9 subtelomeric (9pter, green) and chromosome 9 pericentromeric heterochromatin (blue) probes. **d** FISH analysis with chromosome 9 subtelomeric (9pter, green) and chromosome 17 centromeric (red) probes

FISH with DNA probe for subtelomeric region17p was performed for the targeted validation of detected CNV. FISH-analysis established that this segment had translocated from the short arm of chromosome 17 (Fig. 3a).

To find out the origin of the identified rearrangement, FISH analysis of the proband’s parents was undertaken. The FISH analysis of the proband’s mother using arm-specific DNA probes for chromosome 17 revealed presence of a fluorescent signal corresponding to the material of the short arm of chromosome 17 on one of homologues of chromosome 9 (Fig. 3b).

The father had a normal FISH results.

In cases of unbalanced translocation, CMA usually shows simultaneously copy number gain and copy number loss of terminal regions of chromosomes involved in the rearrangement. However, no deletion in chromosome 9 could be identified in proband (Fig. 2b).

For additional characterization of derivative chromosome 9, the FISH analysis of the proband’s metaphase spreads was carried out using a combination of whole chromosome 17 painting probe, 9p subtelomeric probe and 9cen probe as a control. Simultaneous hybridization has shown that material of chromosome 17 has been translocated distal to the 9p subtelomere region in derivative chromosome 9 (Fig. 3c).

The FISH analysis of the proband's mother using a 9p subtelomeric DNA probe discovered three fluorescent signals, two of which were observed on chromosomes 9, one of them was stronger than other, and one low-intensity hybridization signal was present on the short arm of chromosome 17 (Fig. 3d). This evidences that the terminal region of chromosome 9 was also involved in the translocation. Thus, the proband’s mother was a carrier of balanced translocation t(9;17) and the imbalance found in the proband is a result of adjacent -I segregation of maternal reciprocal translocation.

## Discussion

Balanced chromosomal translocations are among the most common chromosomal abnormalities in the human general population, carriage prevalence varying between 1/1300 and 1/600. As a rule, carriers of reciprocal translocations have a normal phenotype and at the same time a high risk of infertility, reproductive losses and offsprings with congenital hereditary diseases [7].

Clinical implementation of CMA facilitates high-yield detection of genome-wide imbalances in mentally retarded patients. However, this up-to-date method does not find balanced rearrangements such as balanced translocations, insertions or balanced inversions that can be detected by conventional karyotyping or FISH [8].

Combination of CMA and FISH has allowed to diagnose 10.5 Mb duplication of the short arm of chromosome 17 affecting region 17p13.3p13.1 and establish that the discovered genomic imbalance is a consequence of unbalanced translocation t(9;17) due to malsegregation of maternal balanced translocation. At that, the clinical phenotype of the proband formed not only under the influence of increased number of copies of genes located in region 17p13.3p13.1, but also due to partial deletion of 9p subtelomeric region. Unfortunately, therefore it is impossible to assess the phenotypic effect of such imbalance. On the one hand 9p subtelomeric DNA-probe with a size of 195 kb between RH80320 (proximal) and RH65569 (distal) markers was used. A weak signal of a 9pter on maternal derivative chromosome 17 indicates that the break point on chromosome 9 is located at a distance of at least 350 kb from the telomere. The translocated segment contains 4 OMIM genes, including the *WASHC1, FOXD4, CBWD1,* along with one OMIM Morbid Map gene, *DOCK8* genes. The WASH complex playing a key role in non-neuronal endosomal trafficking by activating Arp2/3, regulates the fission of tubules that serve as transport intermediates during endosome sorting. Diseases associated with *WASHC1* include Wiskott-Aldrich Syndrome and Ritscher-Schinzel Syndrome [9]. *FOXD4* is responsible for speech and language development and dysregulation of this gene may leads to speech delay seen in our patient. This gene encodes a member of the forkhead/winged helix-box (FOX) family of transcription factors. Haploinsufficiency of *FOXD4* may cause dosage imbalance in its target genes, which leads to abnormal development [10]. *CBWD1* is a protein coding gene, but there is no diseases associated with mutations of *CBWD1* gene [11]*.* Heterozygous deletions and mutations of *DOCK8* are associated with autosomal recessive hyper-IgE recurrent infection syndrome (OMIM 243700). *DOCK8* also plays an important role in brain development and cognitive function, which are associated with mental or behavioral disorders [12]. Heterozygous disorder of *DOCK8* due to chromosomal deletion or a translocation breakpoint is related to autosomal dominant mental retardation 2 (OMIM 614113) [13].

**Fig. 4:**
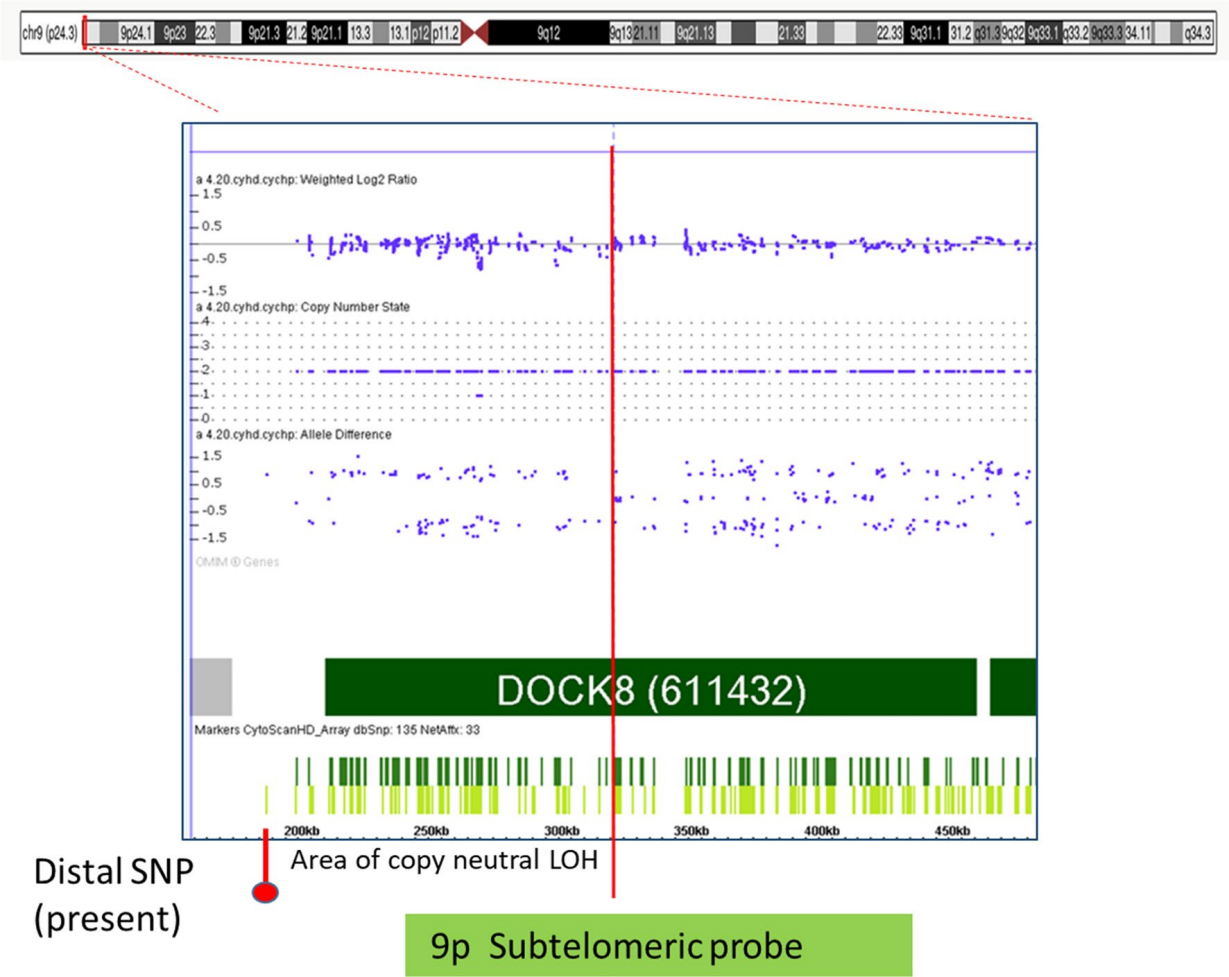
Schematic diagram showing the 9pter region

On the other hand, CMA of the proband showed that the last distal SNP (S-3UNQB) located at chr9:192129 is present. Therefore, the deletion with a size of 350 kb should be detected. Nevertheless, CMA did not show a 9p deletion, but allele difference plot and B-allele frequency (BAF) plot showed two allele tracts instead of three, indicating a presence of a single allele (Fig. 4). A possible explanation for such CMA finding could be duplication of the corresponding region on the paternal homologue of chromosome 9. Indeed, according to DGV, duplications involving these genes are present at unaffected individuals. Previous studies on *DOCK8* duplications are limited and they indicate that rare CNVs might be benign for most patients who inherit from unaffected parents [14, 15]. Unfortunately, the proband's father was unavailable for CMA to prove our assumption.

To date, just a few patients with duplication of region pter → p13.1 of chromosome 17 have been described. In all cases, the diagnosed genomic imbalance was a consequence of malsegregation of the parent’s reciprocal translocation [16–19]. The patients had a complex phenotype due to a combination of partial trisomy of the short arm of chromosome 17 and partial monosomy of another chromosome involved in the rearrangement. Just one case similar to ours was described when 17p13.1p13.3 duplications due to maternal translocation t(17;19) was detected in a fetus with intrauterine growth retardation [20].

In our patient, terminal 10.5 Mb duplication of the short arm of chromosome 17 affected 315 genes, 55 of which are OMIM-morbid ones, and included 8 genes of the critical region of Miller-Dieker syndrome: *PRP8 (607300), RILP (607848), SREC (607873), PITPNA (600174), SKIP (603055), MYO1C (606538), CRK (164762) and YWHAE (605066).*

Duplication of this region (involving both YWHAE and LIS1 genes) is associated with a variable clinical phenotype that typically includes structural brain abnormalities (involving the corpus callosum, cerebellar vermis, and cranial base), hypotonia, intellectual disability, a relatively distinct facial phenotype, and other variable findings [21].

Curry et al. [21] reviewed 34 individuals from 21 families with duplication 17p13.3 associated with heterogeneous breakpoints, and found several overlapping phenotypes, which were divided into group 1 for telomeric duplications and group 2 for duplications, including larger regions of duplication. Signs associated with telomeric duplications of 17p13.3 (Group 1) included early developmental delay, mild to moderate intellectual disability, increased autism, hypotension and moderately myopathic facial features in infants and young children, mild but characteristic facial dysmorphic features at an older age and occasional mild malformations of the brain involving the corpus callosum, cerebellum, and posterior fossa. Signs associated with a larger 17p13.3 duplication (group 2) also included more striking facial dysmorphic features at an older age, consisting of a long face, small mouth, protruding jaw and pointed chin, and cerebellar and posterior fossa malformations, that are more persistent and severe than those found with smaller duplications. However, none were pathognomonic or likely to allow recognition of this genomic disorder on clinical grounds alone.

It was surmised that duplication *YWHAE* might affect the development and maturing of the neuronal network and is associated with a mild developmental delay or mental retardation and facial dysmorphism while duplication *PAFAH1B1* leading to its overexpression is associated with a moderate or severe developmental delay and structural abnormalities of the brain [1].

At present, only 13 patients with 17p13.3 duplications containing both gene *PAFAH1B1* and gene *YWHAE* are registered [1]. All of them, like our patient, have developmental psychomotor and speech delay verbal retardation and characteristic specific facial features such as hypertelorism, short nose, small mouth (Table 1).

**Table 1:** Comparison of 17p segmental duplication clinical features among our case and others previously published

Paper	[22]	[3]	[3]	[3]	[4]	[21]	[21]	[21]	[23]	[7]	[24]	[24]	[24]	Present Study
Patient reference	Patient 7	Patient 1	Patient 2	Patient 3	Patient 10	Patient 12	Patient 13	Patient 15	Patient 1	Patient 1	Patient 1	Patient 2	Patient 3	Patient 1
Size of duplication, Mb	3.6	1.8	3	4	2	3.4	2.78	2.16	5.7	3.9	4.2	4.2	4.2	10.5
Age at diagnosis, years	10	14	1	1	6.5	28	1.1	14	1.4	4	40	39	31	2.3
Gender	F	M	F	M	M	F	M	F	F	M	M	F	F	F
Birth height, cm	53	53	NA	50	Normal	NA	NA	NA	55	51	NA	NA	NA	52
Birth weight, g	3060	3350	4200	3380	Normal	NA	NA	NA	2680	3200	3200	2800	2700	3350
Cranio-facial dysmorphism hypotonic face	–	+	+	+	NA	+	+	NA	+	−	NA	NA	NA	+
High forehead	–	+	+	+	NA	NA	+	NA	+	−	NA	NA	NA	+
Hypertelorism	–	+	+	+	–	–	–	–	+	−	+	+	+	+
Broad nasal bridge	–	+	+	+	–	NA	+	NA	+	−	+	+	+	+
Small mouth	Normal	Normal	+	+	Prominent cupid bow	+	+	+	+	−	+	+	+	+
Low-set-ears	–	+	+	–	NA	NA	+	NA	+	−	+	+	+	+
Triangular chin	NA	–	+	+	+	+	+	+	+	−	+	+	+	−
Neck appearance	Normal	Normal	Short	Short	Normal	NA	NA	NA	NA	−	NA	NA	NA	Short
Neurological features hypotonia	–	+	+	+	+	NA	–	+	NA	+	+	+	+	−
Delayed mental development	+	+	+	+	–	LD	Mild LD	Mild LD	+	+	+	+	+	+
Delayed motor development	+	+	+	+	–	+	NA	+	+	+	+	+	+	+
Abnormal behavior	+	+	+	+	Autism	+	–	–	−	Autism	−	−	−	−
Brain imaging results	Reduced brain size, corpus callosum hypoplasia, cerebellar agenesis	Normal	NA	Dilated lateral ventricles/corpus callosum agenesis	NA	NA	Thin corpus callosum, cerebellar vermis hypoplasia	NA	Normal	Polymicrogyria	NA	NA	NA	NA

In spite of extended duplication (10.5 Mb) and a large number of genes involved therein, the proband`s phenotype largely matched the clinical features typical for 17p13 duplication syndrome (OMIM # 613215, chromosome 17p13.3, centromeric, duplication syndrome). The common features included psychomotor and speech delay, prominent hypertelorism, posteriorly rotated ears, a short nose, small mouth, hypotonia.

Gene *BHLHA9* located in the critical region of 17p13.3 microduplication syndrome, but outside the critical region of Miller–Dieker syndrome was associated with split-hand/foot malformation developmental defects of extremeties [25, 26]. OMIM database annotates the syndrome of duplication of telomeric region 17p13.3. The syndrome is characterized by significant penetrance and high expressivity. In our case, hand/foot malformations with long bone deficiency were not noted. According to literature, 17p13.3 duplications with hand/foot malformation affliction of extremities were relatively small in size (263 kb approximately), involved gene *BHLHA9*, with breakpoints in the regions of genes *ABR-TUSC5*. On the contrary, in the patients whose extremities were not afflicted but who suffered from mental retardation, 17p13.3 duplications were large in size (1.1 Mb on average) and did not break the region of genes *ABR-TUSC5*. This suggests that disturbance of neighboring, supposedly regulatory, elements (for example, in region *ABR-TUSC5*) of gene *BHLHA9* might be a complementary factor promoting occurrence of hand/foot malformations with long bone deficiency [27].

## Conclusions

In this article, we described a new case of duplication 17p13.3p13.1 due to maternal balanced translocation t(9;17). Major contribution to the abnormal phenotype is duplication of 17p13. Clinical features in our patient including psychomotor and speech delay, marked hypertelorism, back-turned ears, short nose, small mouth, and hypotonia are typical for the 17p13 duplication syndrome. Large duplication of 17p13 is usually indicates a more severe phenotype than those found with smaller duplications, but more striking dysmorphic findings appear at older ages. The reported patient is a girl 2 years 3 months old and only further monitoring of the proband’s clinical features and additional instrumental studies imaging (MRI) will confirm this statement. Moreover, in fact our patient also had a deletion of subtelomeric region 9p which was not detected by CMA but could contribute to developmental delay or mental retardation. This additional genomic imbalances may not be directly responsible for the diagnosis, but it`s effect in the phenotype should be considered.

CMA allowed to identify the unbalanced fragment responsible for occurrence of clinical features in the patient. But when a copy number gain is detected by CMA, it is impossible to determine where in the genome the additional material resides using the array data alone. FISH analysis is the preferred method to identify both the location and the copy number of the genomic segments. The importance of follow-up parental studies is also illustrated in this study. The proband’s mother was diagnosed as a carrier of balanced chromosomal translocation t(9;17), associated with the risk of formation of gametes with 17p duplication or deletion. If CMA was perform alone one would assume that the terminal duplication was a sporadic event with a low recurrence rate. Since the results of the parental studies demonstrated that the duplication was inherited from a parental balanced rearrangement, the recurrence risk and genetic counseling for this family were dramatically altered.

Thereby CMA followed by targeted FISH allows a most comprehensive characterization of an identified chromosomal/genomic imbalance and also provision of an adequate medical and genetic counseling for a family taking into account a balanced chromosomal rearrangement.

## Data Availability

All data generated or analyzed during this study available from the corresponding author on reasonable request.

## Abbreviations

CMA: Chromosomal microarray analysis
CNVs: Copy number variants
OMIM: Online Mendelian inheritance in man
ISCN 2016: International system for human cytogenetic nomenclature
DGV: Database of genomic variants
NCBI: National center for biotechnology information
SD: Standard deviations
